# Editorial Expression of Concern: Response of human pancreatic cancer cell xenografts to tetraiodothyroacetic acid nanoparticles

**DOI:** 10.1007/s12672-023-00668-5

**Published:** 2023-05-22

**Authors:** Murat Yalcin, Hung-Yun Lin, Thangirala Sudha, Dhruba J. Bharali, Ran Meng, Heng-Yuan Tang, Faith B. Davis, Steven C. Stain, Paul J. Davis, Shaker A. Mousa

**Affiliations:** 1 Pharmaceutical Research Institute, Albany College of Pharmacy and Health Sciences, 1 Discovery Drive, Rensselaer, Albany, NY 12208 USA; 2 Department of Physiology, Veterinary Medicine Faculty, Uludag University, Gorukle, 16059 Bursa, Turkey; 3 Institute of Cancer Biology and Drug Discovery, Taipei Medical University, Taipei, Taiwan; 4 Department of Medicine, Albany Medical College, Albany, NY 12208 USA; 5 Department of Surgery, Albany Medical College, Albany, NY 12208 USA

**Editorial Expression of Concern: HORM CANC (2013) 4:176–185** 10.1007/s12672-013-0137-y

The Editor-in-Chief would like to alert readers that concerns have been raised regarding Fig. 2 presented in this article. Similarities have been noted in lanes 2 and 6 of Fig. 2B. Readers are advised to interpret the results with caution. Authors have not responded to any correspondence from the publisher about this editorial expression of concern.

